# *catena*-Poly[[[tetra­aqua­cobalt(II)]-μ-4,4′-bipyridine-κ^2^ *N*:*N*′] pyridine-3,5-dicarboxyl­ate trihydrate]

**DOI:** 10.1107/S1600536811023816

**Published:** 2011-06-25

**Authors:** Xian-Dong Zhu

**Affiliations:** a College of Biological and Chemical Engineering, Anhui Polytechnic University, Wuhu 241000, People’s Republic of China

## Abstract

The crystal structure of the title compound, {[Co(C_10_H_8_N_2_)(H_2_O)_4_](C_7_H_3_NO_4_)·3H_2_O}_*n*_, consists of Co^II^ polymeric complex cations, uncoordinated pyridine-3,5-dicarboxyl­ate anions and lattice water mol­ecules. The Co^II^ cation is coordinated by two N atoms from two 4,4′-bipyridine ligands and four water mol­ecules in a distorted octa­hedral geometry. The 4,4′-bipyridine ligands bridge Co cations, forming a polymeric chain running along the *b* axis. The two pyridine rings of the 4,4′-biyridine are twisted to each other by a dihedral angle of 8.95 (9)°. Extensive O—H⋯O hydrogen bonding network is present in the crystal structure.

## Related literature

For the utility of 4,4′-bipyridine in assembling metal-organic frameworks, see: Briadha & Fujita (2001 ▶). For related complexes, see: Li *et al.* (2004 ▶); Zhang & Zhu (2005 ▶). For the synthesis, see: Whitfield *et al.* (2001 ▶).
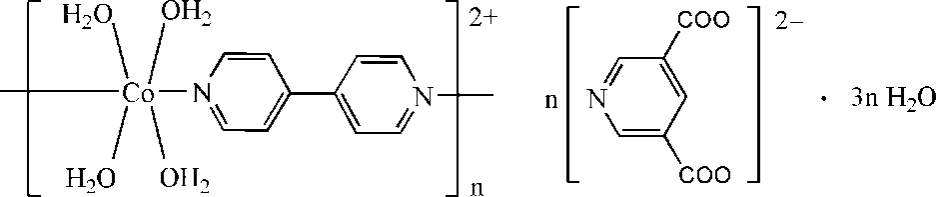


## Experimental

### 

#### Crystal data

[Co(C_10_H_8_N_2_)(H_2_O)_4_](C_7_H_3_NO_4_)·3H_2_O
*M* *_r_* = 506.33
Triclinic, 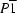

*a* = 7.0053 (18) Å
*b* = 11.449 (3) Å
*c* = 14.077 (4) Å
α = 105.352 (4)°
β = 92.837 (4)°
γ = 94.624 (2)°
*V* = 1082.2 (5) Å^3^
*Z* = 2
Mo *K*α radiation
μ = 0.86 mm^−1^
*T* = 293 K
0.50 × 0.40 × 0.20 mm

#### Data collection

Bruker SMART 1000 CCD diffractometer
Absorption correction: multi-scan (*SADABS*; Bruker, 2001 ▶) *T*_min_ = 0.669, *T*_max_ = 0.842
8332 measured reflections
4863 independent reflections
4345 reflections with *I* > 2σ(*I*)
*R*_int_ = 0.012

#### Refinement

*R*[*F* ^2^ > 2σ(*F* ^2^)] = 0.033
*wR*(*F* ^2^) = 0.118
*S* = 0.96
4863 reflections
289 parameters
H-atom parameters constrained
Δρ_max_ = 0.51 e Å^−3^
Δρ_min_ = −0.28 e Å^−3^

### 

Data collection: *SMART* (Bruker, 2007 ▶); cell refinement: *SAINT* (Bruker, 2007 ▶); data reduction: *SAINT*; program(s) used to solve structure: *SHELXTL* (Sheldrick, 2008 ▶); program(s) used to refine structure: *SHELXTL*; molecular graphics: *SHELXTL*; software used to prepare material for publication: *SHELXTL*.

## Supplementary Material

Crystal structure: contains datablock(s) I, global. DOI: 10.1107/S1600536811023816/xu5203sup1.cif

Structure factors: contains datablock(s) I. DOI: 10.1107/S1600536811023816/xu5203Isup2.hkl

Additional supplementary materials:  crystallographic information; 3D view; checkCIF report

## Figures and Tables

**Table 1:** Selected bond lengths (Å)

Co1—O1*W*	2.0898 (12)
Co1—O2*W*	2.0764 (14)
Co1—O3*W*	2.1245 (13)
Co1—O4*W*	2.0709 (14)
Co1—N1	2.1692 (14)
Co1—N2^i^	2.1543 (14)

Symmetry code: (i) 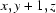.

**Table 2:** Hydrogen-bond geometry (Å, °)

*D*—H⋯*A*	*D*—H	H⋯*A*	*D*⋯*A*	*D*—H⋯*A*
O1*W*—H1*A*⋯O2^ii^	0.85	1.84	2.6813 (19)	173
O1*W*—H1*B*⋯O1^iii^	0.85	1.96	2.780 (2)	162
O2*W*—H2*A*⋯O1	0.85	1.91	2.760 (2)	176
O2*W*—H2*B*⋯O6*W*	0.85	1.87	2.707 (2)	168
O3*W*—H3*A*⋯O1^iii^	0.85	2.12	2.897 (2)	152
O3*W*—H3*B*⋯O3^iv^	0.85	1.89	2.7408 (19)	176
O4*W*—H4*A*⋯O5*W*	0.85	1.82	2.665 (3)	171
O4*W*—H4*B*⋯O7*W*	0.85	1.90	2.734 (2)	168
O5*W*—H5*A*⋯O4^i^	0.85	1.90	2.747 (3)	172
O5*W*—H5*B*⋯O4^v^	0.85	2.19	2.856 (3)	135
O6*W*—H6*A*⋯N3^i^	0.85	1.98	2.829 (2)	173
O6*W*—H6*B*⋯O2^vi^	0.85	1.99	2.830 (2)	172
O7*W*—H7*A*⋯O3^v^	0.85	1.98	2.828 (2)	175
O7*W*—H7*B*⋯O3	0.85	1.96	2.800 (2)	168

Symmetry codes: (i) 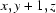; (ii) 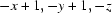; (iii) 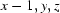; (iv) 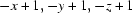; (v) 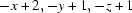; (vi) 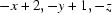.

**Fig. 1.**
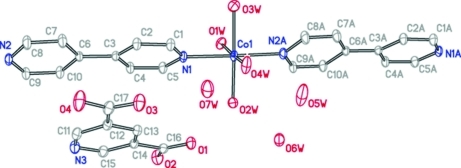
A fragment of one-dimensional chain structure of the title compound showing the atom numbering scheme. Displacement ellipsoids are drawn at the 30% probability level for non-H atoms.

## supplementary crystallographic information

### Comment

The utility of linear bifunctional ligands, such as 4,4'-bipyridine, has been widely explored in the field of the crystal engineering of metal-organic frameworks (Briadha *et al.*, 2001). Recently, we are interested in the assembly of new compounds which contain not only 4,4'-bipyridine ligand but also carboxylate groups in the crystal structure. In this paper, we report the synthesis and crystal structure of the title compound.

In the title compound, the cation shows a slightly distorted octahedral coordination environment composed of a six-coordinated Co(II) center. The 4,4'-bipyridine units bridge the Co(II) atoms directly to form a one-dimensional chain; similar to a Co^II^ complex (Li *et al.*, 2004) and and a Ni^II^ complex (Zhang & Zhu, 2005) reported previously. The pyridine-3,5-dicarboxylate anion does not take part in coordination, but acts as a charge balance with two deprotonated carboxylate groups, and supplies hydrogen-bonding donor and acceptors. O—H···O and N—H···O hydrogen-bonds exist between uncoordinated anion, uncoordinated water and coordinated water molecules, which connect the one-dimensional chain into three-dimensional supramolecular network.

### Experimental

A mixture of Co(NO_3_)_2_.6H_2_O (0.064 g, 0.2 mmol), 4,4-bipyridine (0.034 g, 0.2 mmol), pyridine-3,5-dicarboxylic acid (0.034 g, 0.2 mmol), NaOH (0.008 g, 0.2 mmol) in water (10 ml) was sealed in a 25 ml Teflon-lined stainless steel autoclave. The mixture was heated at 423 K for 72 h, then slowly cooled to room temperature during 48 h. Two kinds of crystals were obtained from the reaction mixture. One is purple and needle shaped, which structure was reported by Whitfield *et al.* (2001); the other one is red and prism shaped, the structure is reported here.

### Refinement

H atoms bonded to C atoms were placed in calculated positions with C—H distances of 0.95 Å and included in the refinement with a riding-mode approximation with *U*_iso_(H) = 1.2U_eq_(C). Water H atoms were located in a difference Fourier map but they were treated as riding on their parent atoms with O—H = 0.85 Å, H—H = 1.39 Å, and U_iso_(H) = 1.5U_eq_(O).

### Crystal data

**Table:**

[Co(C_10_H_8_N_2_)(H_2_O)_4_](C_7_H_3_NO_4_)·3H_2_O	*Z* = 2
*M**_r_* = 506.33	*F*(000) = 526
Triclinic, *P*1	*D*_x_ = 1.554 Mg m^−^^3^
Hall symbol: -P 1	Mo *K*α radiation, λ = 0.71073 Å
*a* = 7.0053 (18) Å	Cell parameters from 2942 reflections
*b* = 11.449 (3) Å	θ = 2.1–27.5°
*c* = 14.077 (4) Å	µ = 0.86 mm^−^^1^
α = 105.352 (4)°	*T* = 293 K
β = 92.837 (4)°	Prism, red
γ = 94.624 (2)°	0.50 × 0.40 × 0.20 mm
*V* = 1082.2 (5) Å^3^	

### Data collection

**Table:**

Bruker SMART 1000 CCD diffractometer	4863 independent reflections
Radiation source: fine-focus sealed tube	4345 reflections with *I* > 2σ(*I*)
graphite	*R*_int_ = 0.012
φ and ω scans	θ_max_ = 27.5°, θ_min_ = 2.1°
Absorption correction: multi-scan (*SADABS*; Bruker, 2001)	*h* = −9→8
*T*_min_ = 0.669, *T*_max_ = 0.842	*k* = −14→14
8332 measured reflections	*l* = −15→18

### Refinement

**Table:**

Refinement on *F*^2^	Primary atom site location: structure-invariant direct methods
Least-squares matrix: full	Secondary atom site location: difference Fourier map
*R*[*F*^2^ > 2σ(*F*^2^)] = 0.033	Hydrogen site location: inferred from neighbouring sites
*wR*(*F*^2^) = 0.118	H-atom parameters constrained
*S* = 0.96	*w* = 1/[σ^2^(*F*_o_^2^) + (0.1*P*)^2^] where *P* = (*F*_o_^2^ + 2*F*_c_^2^)/3
4863 reflections	(Δ/σ)_max_ = 0.001
289 parameters	Δρ_max_ = 0.51 e Å^−^^3^
0 restraints	Δρ_min_ = −0.28 e Å^−^^3^

### Special details

**Table:**

Geometry. All e.s.d.'s (except the e.s.d. in the dihedral angle between two l.s. planes) are estimated using the full covariance matrix. The cell e.s.d.'s are taken into account individually in the estimation of e.s.d.'s in distances, angles and torsion angles; correlations between e.s.d.'s in cell parameters are only used when they are defined by crystal symmetry. An approximate (isotropic) treatment of cell e.s.d.'s is used for estimating e.s.d.'s involving l.s. planes.
Refinement. Refinement of *F*^2^ against ALL reflections. The weighted *R*-factor *wR* and goodness of fit *S* are based on *F*^2^, conventional *R*-factors *R* are based on *F*, with *F* set to zero for negative *F*^2^. The threshold expression of *F*^2^ > σ(*F*^2^) is used only for calculating *R*-factors(gt) *etc*. and is not relevant to the choice of reflections for refinement. *R*-factors based on *F*^2^ are statistically about twice as large as those based on *F*, and *R*- factors based on ALL data will be even larger.

### Fractional atomic coordinates and isotropic or equivalent isotropic displacement parameters (Å^2^)

**Table:**

	*x*	*y*	*z*	*U*_iso_*/*U*_eq_	
Co1	0.38876 (3)	0.627012 (17)	0.230821 (15)	0.02719 (11)	
O1	0.88432 (18)	0.45647 (11)	0.17193 (9)	0.0357 (3)	
O2	0.8709 (2)	0.31057 (12)	0.02985 (9)	0.0457 (3)	
O3	0.8583 (2)	0.32631 (11)	0.48551 (9)	0.0457 (3)	
O4	0.8299 (3)	0.12902 (13)	0.47420 (10)	0.0588 (4)	
N1	0.3885 (2)	0.43762 (12)	0.23107 (10)	0.0289 (3)	
N2	0.3821 (2)	−0.18489 (12)	0.23179 (10)	0.0302 (3)	
N3	0.8803 (2)	0.03959 (13)	0.17180 (11)	0.0377 (3)	
C1	0.3827 (3)	0.40339 (14)	0.31469 (12)	0.0331 (4)	
H1	0.3823	0.4648	0.3751	0.040*	
C2	0.3773 (3)	0.28345 (14)	0.31811 (12)	0.0313 (3)	
H2	0.3750	0.2645	0.3798	0.038*	
C3	0.3752 (2)	0.19091 (13)	0.23145 (11)	0.0252 (3)	
C4	0.3781 (3)	0.22635 (14)	0.14371 (12)	0.0312 (3)	
H4	0.3747	0.1666	0.0821	0.037*	
C5	0.3860 (3)	0.34851 (15)	0.14656 (12)	0.0326 (3)	
H5	0.3898	0.3704	0.0861	0.039*	
C6	0.3733 (2)	0.06074 (13)	0.23155 (11)	0.0261 (3)	
C7	0.3486 (3)	0.02297 (15)	0.31603 (13)	0.0380 (4)	
H7	0.3283	0.0807	0.3761	0.046*	
C8	0.3531 (3)	−0.09846 (15)	0.31378 (13)	0.0402 (4)	
H8	0.3349	−0.1217	0.3729	0.048*	
C9	0.4037 (3)	−0.14903 (14)	0.14960 (13)	0.0331 (4)	
H9	0.4233	−0.2086	0.0905	0.040*	
C10	0.3990 (3)	−0.02929 (14)	0.14642 (12)	0.0326 (4)	
H10	0.4135	−0.0088	0.0859	0.039*	
C11	0.8681 (3)	0.07055 (16)	0.26959 (13)	0.0338 (4)	
H11	0.8642	0.0075	0.3020	0.041*	
C12	0.8609 (2)	0.18844 (15)	0.32658 (11)	0.0288 (3)	
C13	0.8678 (2)	0.28087 (14)	0.27885 (11)	0.0274 (3)	
H13	0.8659	0.3633	0.3155	0.033*	
C14	0.8773 (2)	0.25103 (14)	0.17736 (11)	0.0262 (3)	
C15	0.8837 (2)	0.12965 (15)	0.12731 (12)	0.0327 (3)	
H15	0.8908	0.1093	0.0577	0.039*	
C16	0.8777 (2)	0.34712 (14)	0.12216 (11)	0.0288 (3)	
C17	0.8465 (3)	0.21575 (15)	0.43678 (12)	0.0356 (4)	
O1W	0.19781 (18)	0.56791 (11)	0.10504 (8)	0.0357 (3)	
H1A	0.1813	0.6024	0.0591	0.043*	
H1B	0.0898	0.5339	0.1132	0.043*	
O2W	0.6205 (2)	0.61273 (12)	0.14338 (12)	0.0472 (3)	
H2A	0.7040	0.5672	0.1545	0.057*	
H2B	0.6924	0.6702	0.1320	0.057*	
O3W	0.14285 (19)	0.63665 (11)	0.31403 (9)	0.0380 (3)	
H3A	0.0450	0.5866	0.2905	0.046*	
H3B	0.1477	0.6495	0.3764	0.046*	
O4W	0.5585 (2)	0.68048 (12)	0.36252 (11)	0.0510 (4)	
H4A	0.6317	0.7466	0.3793	0.061*	
H4B	0.6193	0.6310	0.3850	0.061*	
O5W	0.8025 (3)	0.87961 (16)	0.43207 (17)	0.0859 (7)	
H5A	0.7997	0.9560	0.4422	0.103*	
H5B	0.9151	0.8574	0.4258	0.103*	
O6W	0.8252 (2)	0.78607 (12)	0.08166 (12)	0.0575 (4)	
H6A	0.8500	0.8610	0.1118	0.069*	
H6B	0.9238	0.7623	0.0522	0.069*	
O7W	0.7707 (2)	0.55000 (13)	0.45807 (11)	0.0552 (4)	
H7A	0.8792	0.5902	0.4781	0.066*	
H7B	0.7806	0.4780	0.4623	0.066*	

### Atomic displacement parameters (Å^2^)

**Table:**

	*U*^11^	*U*^22^	*U*^33^	*U*^12^	*U*^13^	*U*^23^
Co1	0.03388 (16)	0.01625 (15)	0.03333 (15)	0.00209 (9)	0.00055 (10)	0.01041 (10)
O1	0.0457 (7)	0.0266 (6)	0.0370 (6)	0.0041 (5)	0.0037 (5)	0.0123 (5)
O2	0.0725 (10)	0.0417 (7)	0.0278 (6)	0.0085 (7)	0.0049 (6)	0.0165 (5)
O3	0.0727 (10)	0.0329 (7)	0.0310 (6)	−0.0010 (6)	0.0004 (6)	0.0103 (5)
O4	0.1060 (13)	0.0368 (7)	0.0369 (7)	−0.0019 (8)	0.0017 (7)	0.0190 (6)
N1	0.0356 (7)	0.0161 (6)	0.0364 (7)	0.0018 (5)	0.0015 (5)	0.0103 (5)
N2	0.0375 (7)	0.0179 (6)	0.0378 (7)	0.0037 (5)	0.0024 (5)	0.0120 (5)
N3	0.0482 (9)	0.0256 (7)	0.0395 (8)	0.0035 (6)	0.0054 (6)	0.0087 (6)
C1	0.0448 (10)	0.0211 (7)	0.0341 (8)	0.0023 (7)	0.0019 (7)	0.0090 (6)
C2	0.0421 (9)	0.0221 (7)	0.0312 (8)	0.0024 (6)	0.0012 (6)	0.0104 (6)
C3	0.0248 (7)	0.0185 (7)	0.0340 (8)	0.0025 (5)	0.0017 (5)	0.0102 (6)
C4	0.0418 (9)	0.0195 (7)	0.0329 (8)	0.0037 (6)	0.0031 (6)	0.0081 (6)
C5	0.0435 (9)	0.0236 (8)	0.0334 (8)	0.0040 (7)	0.0029 (6)	0.0123 (6)
C6	0.0255 (7)	0.0189 (7)	0.0357 (8)	0.0029 (5)	0.0012 (6)	0.0107 (6)
C7	0.0607 (12)	0.0220 (8)	0.0345 (9)	0.0088 (7)	0.0112 (8)	0.0104 (6)
C8	0.0630 (12)	0.0240 (8)	0.0388 (9)	0.0096 (8)	0.0119 (8)	0.0145 (7)
C9	0.0446 (9)	0.0183 (7)	0.0361 (8)	0.0038 (6)	0.0028 (7)	0.0069 (6)
C10	0.0461 (10)	0.0213 (7)	0.0326 (8)	0.0039 (7)	0.0020 (7)	0.0112 (6)
C11	0.0401 (9)	0.0274 (8)	0.0372 (8)	0.0005 (7)	0.0017 (7)	0.0157 (7)
C12	0.0305 (8)	0.0279 (8)	0.0290 (7)	−0.0004 (6)	−0.0008 (6)	0.0111 (6)
C13	0.0310 (8)	0.0235 (7)	0.0290 (7)	0.0017 (6)	0.0016 (6)	0.0098 (6)
C14	0.0252 (7)	0.0268 (8)	0.0293 (7)	0.0025 (6)	0.0029 (5)	0.0119 (6)
C15	0.0385 (9)	0.0302 (8)	0.0297 (8)	0.0018 (7)	0.0037 (6)	0.0090 (6)
C16	0.0289 (8)	0.0298 (8)	0.0314 (8)	0.0035 (6)	0.0041 (6)	0.0139 (6)
C17	0.0445 (10)	0.0323 (8)	0.0312 (8)	−0.0019 (7)	−0.0030 (7)	0.0135 (7)
O1W	0.0410 (7)	0.0348 (6)	0.0350 (6)	−0.0028 (5)	−0.0033 (5)	0.0192 (5)
O2W	0.0391 (7)	0.0313 (6)	0.0813 (10)	0.0112 (5)	0.0197 (6)	0.0279 (7)
O3W	0.0427 (7)	0.0393 (7)	0.0297 (6)	−0.0022 (5)	0.0051 (5)	0.0066 (5)
O4W	0.0613 (9)	0.0267 (6)	0.0630 (9)	−0.0064 (6)	−0.0266 (7)	0.0171 (6)
O5W	0.0886 (14)	0.0368 (9)	0.1268 (17)	−0.0160 (9)	−0.0430 (12)	0.0281 (10)
O6W	0.0705 (10)	0.0318 (7)	0.0688 (10)	0.0014 (7)	0.0318 (8)	0.0069 (6)
O7W	0.0640 (10)	0.0390 (7)	0.0635 (9)	0.0050 (7)	−0.0161 (7)	0.0190 (7)

### Geometric parameters (Å, °)

**Table:**

Co1—O1W	2.0898 (12)	C7—H7	0.9500
Co1—O2W	2.0764 (14)	C8—H8	0.9500
Co1—O3W	2.1245 (13)	C9—C10	1.386 (2)
Co1—O4W	2.0709 (14)	C9—H9	0.9500
Co1—N1	2.1692 (14)	C10—H10	0.9500
Co1—N2^i^	2.1543 (14)	C11—C12	1.382 (2)
O1—C16	1.258 (2)	C11—H11	0.9500
O2—C16	1.252 (2)	C12—C13	1.395 (2)
O3—C17	1.264 (2)	C12—C17	1.509 (2)
O4—C17	1.242 (2)	C13—C14	1.385 (2)
N1—C1	1.337 (2)	C13—H13	0.9500
N1—C5	1.345 (2)	C14—C15	1.387 (2)
N2—C9	1.337 (2)	C14—C16	1.505 (2)
N2—C8	1.343 (2)	C15—H15	0.9500
N3—C11	1.336 (2)	O1W—H1A	0.8501
N3—C15	1.340 (2)	O1W—H1B	0.8499
C1—C2	1.384 (2)	O2W—H2A	0.8501
C1—H1	0.9500	O2W—H2B	0.8499
C2—C3	1.386 (2)	O3W—H3A	0.8500
C2—H2	0.9500	O3W—H3B	0.8500
C3—C4	1.399 (2)	O4W—H4A	0.8498
C3—C6	1.490 (2)	O4W—H4B	0.8499
C4—C5	1.385 (2)	O5W—H5A	0.8500
C4—H4	0.9500	O5W—H5B	0.8500
C5—H5	0.9500	O6W—H6A	0.8499
C6—C7	1.383 (2)	O6W—H6B	0.8499
C6—C10	1.389 (2)	O7W—H7A	0.8501
C7—C8	1.385 (2)	O7W—H7B	0.8499
			
O4W—Co1—O2W	94.18 (7)	N2—C8—H8	118.5
O4W—Co1—O1W	174.82 (5)	C7—C8—H8	118.5
O2W—Co1—O1W	90.62 (6)	N2—C9—C10	123.27 (16)
O4W—Co1—O3W	88.56 (6)	N2—C9—H9	118.4
O2W—Co1—O3W	177.17 (5)	C10—C9—H9	118.4
O1W—Co1—O3W	86.62 (5)	C9—C10—C6	120.15 (15)
O4W—Co1—N2^i^	89.64 (5)	C9—C10—H10	119.9
O2W—Co1—N2^i^	90.37 (5)	C6—C10—H10	119.9
O1W—Co1—N2^i^	92.30 (5)	N3—C11—C12	124.18 (15)
O3W—Co1—N2^i^	90.39 (5)	N3—C11—H11	117.9
O4W—Co1—N1	90.65 (5)	C12—C11—H11	117.9
O2W—Co1—N1	90.96 (5)	C11—C12—C13	117.76 (15)
O1W—Co1—N1	87.30 (5)	C11—C12—C17	120.90 (14)
O3W—Co1—N1	88.27 (5)	C13—C12—C17	121.34 (15)
N2^i^—Co1—N1	178.62 (5)	C14—C13—C12	119.21 (15)
C1—N1—C5	116.80 (14)	C14—C13—H13	120.4
C1—N1—Co1	121.68 (11)	C12—C13—H13	120.4
C5—N1—Co1	121.46 (11)	C13—C14—C15	118.25 (14)
C9—N2—C8	116.73 (14)	C13—C14—C16	121.12 (14)
C9—N2—Co1^ii^	121.19 (11)	C15—C14—C16	120.63 (14)
C8—N2—Co1^ii^	122.07 (11)	N3—C15—C14	123.56 (15)
C11—N3—C15	117.02 (15)	N3—C15—H15	118.2
N1—C1—C2	123.67 (15)	C14—C15—H15	118.2
N1—C1—H1	118.2	O2—C16—O1	125.59 (15)
C2—C1—H1	118.2	O2—C16—C14	116.60 (14)
C3—C2—C1	120.00 (15)	O1—C16—C14	117.81 (14)
C3—C2—H2	120.0	O4—C17—O3	124.16 (16)
C1—C2—H2	120.0	O4—C17—C12	118.32 (16)
C2—C3—C4	116.44 (14)	O3—C17—C12	117.48 (14)
C2—C3—C6	121.95 (15)	Co1—O1W—H1A	127.7
C4—C3—C6	121.61 (15)	Co1—O1W—H1B	115.7
C5—C4—C3	120.09 (15)	H1A—O1W—H1B	107.9
C5—C4—H4	120.0	Co1—O2W—H2A	116.1
C3—C4—H4	120.0	Co1—O2W—H2B	127.7
N1—C5—C4	122.99 (15)	H2A—O2W—H2B	100.5
N1—C5—H5	118.5	Co1—O3W—H3A	118.3
C4—C5—H5	118.5	Co1—O3W—H3B	124.0
C7—C6—C10	116.35 (14)	H3A—O3W—H3B	107.1
C7—C6—C3	122.19 (15)	Co1—O4W—H4A	122.4
C10—C6—C3	121.46 (15)	Co1—O4W—H4B	122.6
C6—C7—C8	120.48 (16)	H4A—O4W—H4B	104.3
C6—C7—H7	119.8	H5A—O5W—H5B	113.0
C8—C7—H7	119.8	H6A—O6W—H6B	107.7
N2—C8—C7	122.99 (16)	H7A—O7W—H7B	106.9

Symmetry codes: (i) *x*, *y*+1, *z*; (ii) *x*, *y*−1, *z*.

### Hydrogen-bond geometry (Å, °)

**Table:**

*D*—H···*A*	*D*—H	H···*A*	*D*···*A*	*D*—H···*A*
O1W—H1A···O2^iii^	0.85	1.84	2.6813 (19)	173
O1W—H1B···O1^iv^	0.85	1.96	2.780 (2)	162
O2W—H2A···O1	0.85	1.91	2.760 (2)	176
O2W—H2B···O6W	0.85	1.87	2.707 (2)	168
O3W—H3A···O1^iv^	0.85	2.12	2.897 (2)	152
O3W—H3B···O3^v^	0.85	1.89	2.7408 (19)	176
O4W—H4A···O5W	0.85	1.82	2.665 (3)	171
O4W—H4B···O7W	0.85	1.90	2.734 (2)	168
O5W—H5A···O4^i^	0.85	1.90	2.747 (3)	172
O5W—H5B···O4^vi^	0.85	2.19	2.856 (3)	135
O6W—H6A···N3^i^	0.85	1.98	2.829 (2)	173
O6W—H6B···O2^vii^	0.85	1.99	2.830 (2)	172
O7W—H7A···O3^vi^	0.85	1.98	2.828 (2)	175
O7W—H7B···O3	0.85	1.96	2.800 (2)	168

Symmetry codes: (iii) −*x*+1, −*y*+1, −*z*; (iv) *x*−1, *y*, *z*; (v) −*x*+1, −*y*+1, −*z*+1; (i) *x*, *y*+1, *z*; (vi) −*x*+2, −*y*+1, −*z*+1; (vii) −*x*+2, −*y*+1, −*z*.

## References

[bb1] Briadha, K. & Fujita, M. (2001). *Chem. Commun.* pp. 15–16.

[bb2] Bruker (2001). *SADABS* Bruker AXS Inc., Madison, Wisconsin, USA.

[bb3] Bruker (2007). *SMART* and *SAINT* Bruker AXS Inc., Madison, Wisconsin, USA.

[bb4] Li, F., Wang, Y., Bi, W., Li, X. & Cao, R. (2004). *Acta Cryst.* E**60**, m1681–m1683.

[bb5] Sheldrick, G. M. (2008). *Acta Cryst.* A**64**, 112–122. 10.1107/S0108767307043930 18156677

[bb6] Whitfield, T., Zheng, L.-M., Wang, X. & Jacobson, A. J. (2001). *Solid State Sci.* **3**, 829–835.

[bb7] Zhang, L.-P. & Zhu, L.-G. (2005). *Acta Cryst.* E**61**, m1264–m1265.

